# Evaluation of the loop-mediated isothermal amplification assay for *Staphylococcus aureus* detection: a systematic review and meta-analysis

**DOI:** 10.1186/s12941-022-00522-6

**Published:** 2022-06-24

**Authors:** Li-Jun Long, Min Lin, Yu-Ran Chen, Xin Meng, Ting-Ting Cui, Ya-Ping Li, Xu-Guang Guo

**Affiliations:** 1 Department of Clinical Laboratory Medicine, The Third Affiliated Hospital of Guangzhou Medical University, Guangzhou, 510150 China; 2 Department of Medical Laboratory Technology, The King Med School of Laboratory Medicine, Guangzhou Medical University, Guangzhou, 511436 China; 3 Department of Traditional Chinese and Western Medicine in Clinical Medicine, The Clinical School of Traditional Chinese and Western Medicine of Guangzhou Medical University, Guangzhou, 511436 China; 4 Department of Medical Imageology, The Third Clinical School of Guangzhou Medical University, Guangzhou, 511436 China; 5 Department of Pediatrics, Pediatrics School, Guangzhou Medical University, Guangzhou, 511436 China; 6 Department of Clinical Medicine, The Second Clinical School of Guangzhou Medical University, Guangzhou, 511436 China; 7 Key Laboratory for Major Obstetric Diseases of Guangdong Province, The Third Affiliated Hospital of Guangzhou Medical University, Guangzhou, 510150 China; 8 Key Laboratory of Reproduction and Genetics of Guangdong Higher Education Institutes, The Third Affiliated Hospital of Guangzhou Medical University, Guangzhou, 510150 China

**Keywords:** *Methicillin-resistant Staphylococcus aureus*, *Staphylococcus aureus*, Loop-mediated isothermal amplification, Detection

## Abstract

**Background:**

*Staphylococcus aureus* can cause many diseases and even death. It’s important to detect *Staphylococcus aureus* rapidly and reliably. The accuracy of a novel test named LAMP in detecting *Staphylococcus aureus* is unclear. Therefore, a systematic review and meta-analysis were conducted to evaluate the accuracy of the LAMP assay for *Staphylococcus aureus* detection.

**Methods:**

Four databases were searched for relevant studies. Meta-DiSc 1.4.0 and Stata 12.0 were used for statistical analysis. At the same time, we used QUADAS-2 to assess the studies we included. Two groups of subgroup analysis were done to differentiate the diagnostic effects of various LAMP tests and in cases of different gold standards.

**Results:**

11 studies were identified and 19 2 × 2 contingency tables were extracted in our study. The results showed that both pooled sensitivity and specificity of the LAMP assay were 99% (95% CI 99–100).

**Conclusion:**

The LAMP assay demonstrated high sensitivity and specificity in diagnosing *Staphylococcus aureus*.

**Supplementary Information:**

The online version contains supplementary material available at 10.1186/s12941-022-00522-6.

## Introduction

*Staphylococcus aureus* (*S. aureus*), including *methicillin-resistant Staphylococcus aureus* (MRSA), is a momentous human pathogen that can produce all kinds of toxins and cause vomiting, diarrhea, gastroenteritis, toxic shock syndrome, and other infectious diseases. It secretes potent enterotoxins, toxic shock syndrome toxin-1, and Panton-Valentine leucocidin [1–3]. With strong virulence, invasiveness, and antibiotic resistance, *S. aureus* has become the primary pathogen of hospital and community-acquired infections [2]. In particular, MRSA has become one of the most crucial epidemiological problems in hospitals worldwide, resulting in a large number of premature deaths [4]. A recent meta-analysis, focusing on the all-cause mortality of *S. aureus* and MRSA, found more than one-third of patients who have *S. aureus* will die within 3 months, and the index of MRSA was higher [5]. Therefore, for diagnosing and treating patients timely, it is vital to establish a reliable and rapid method to detect *S. aureus* and MRSA [6].

The traditional identification methods of *S. aureus* and MRSA in the clinical laboratory are based on the standard phenotypic method, including blood culture, colony morphology, biochemical identification, and antimicrobial susceptibility tests. However, these methods are laborious and time-consuming. On the contrary, polymerase chain reaction (PCR) and real-time PCR detection provide rapid and effective function for the identification of *S. aureus* and MRSA, which become the gold standard at the molecular level [7]. But the relatively expensive thermal cycler limit their applicability in the field [8].

Loop-mediated isothermal amplification (LAMP) was invented by Notomi et al., which is a highly specific method for efficient and rapid amplification of DNA under isothermal conditions. It depends on automated circular strand-substitution DNA synthesis and requires a Bst DNA polymerase with high strand-substitution activity. Besides, a set of internal and external primers for synthesis in LAMP were specially designed to identify all six different sequences on the target DNA. One of the loop primers is needed to bind to the loop structure, which can shorten the reaction time of the LAMP assay. Because its Bst DNA polymerase has isothermal activity and strand-displacement activity, the denaturation steps of the thermocycling reaction can be omitted, thus reducing the technical work and improving the amplification speed [9]. LAMP tests have been developed to rapidly identify all kinds of bacteria, such as *S. aureus*, *Vibrio parahaemolyticus*, *Campylobacter jejuni*, *Campylobacter Coli*, *Leptospira* species, *Salmonella enterica* serovar Typhi and *Escherichia coli *[3].

Compared with PCR, LAMP detection has the same specificity in the identification of *S. aureus* and is cheaper. It is more sensitive and robust in dealing with complex biological samples. Compared with the traditional culture, it is simpler and more efficient with higher sensitivity. At present, LAMP is more suitable for clinical diagnosis, laboratories, and fields with limited resources [3, 10]. In recent years, the commonly used versions used to detect *S. aureus* are eazyplex^®^ MRSAplus (Amplex BioSystems, Giessen, Germany), eazyplex^®^ MRSA (Amplex BioSystems, Giessen, Germany), Loopamp DNA amplification kit (Eiken Chemical Co. Ltd., Tokyo, Japan), LAMP-LFD and m-LAMP-LFB [3]. However, there was no comprehensive evaluation in LAMP for *S. aureus*. Therefore, we evaluate the accuracy of LAMP in the detection of *S. aureus* systematically by combining with previous research data.

## Methods

### Search strategy

Pubmed, Embase, Web of Science, and Cochrane Library were retrieved as of July 30, 2021. The eligible studies were identified according to “*Staphylococcus aureus*”, “*Methicillin-Resistant Staphylococcus aureus*”, “*Vancomycin-Resistant Staphylococcus aureus*”, “*loop-mediated isothermal amplification*”, “*LAMP assay*”, etc. The studies we retrieved were imported into Endnote X9 for management.

### Inclusion and exclusion criteria

The studies which met the following criteria were included: (1) The samples detected and analyzed come from humanity; (2) The number of the specimens is no fewer than ten; (3) Studies use LAMP to detect *S. aureus* and it compares with an appropriate reference standard; (4) The values of 2 × 2 contingency table can be extracted from studies. Editorial, reviews, and conference abstracts are excluded.

### Data abstraction and quality assessment

Two researchers used a pre-designed excel worksheet to extract data from selected articles together. Data includes author, publication year, study design, location, source of specimens, gold standard, type of LAMP, bacterial species, the values of the 2 × 2 contingency table (true positivity (TP), false positivity (FP), false negativity (FN), and true negativity (TN)).

In the meantime, these two researchers evaluated the selected article together using the Quality of Diagnostic Accuracy Studies-2 (QUADAS-2), a recommended tool for appraising studies in systematic reviews for diagnostic accuracy [11]. It is comprised of eleven criteria in four parts, including Patient Selection, Index Test, Reference Standard, and Flow and Timing. Each part was evaluated by different questions and rated as “High”, “Unclear”, and “Low” with risk of bias. In each part, when the answers to all questions for a domain are “yes,” then the risk of bias can be judged low. If any question is answered “no,” potential for bias exists. If insufficient data exists, the answer and risk of bias will be judged unclear [11].

### Statistical analysis

Meta-Disc 1.4.0 and Stata 12.0 were used for statistical analysis. The pooled sensitivity, pooled specificity, positive likelihood ratio (PLR), negative likelihood ratio (NLR), diagnostic odds ratio (DOR), and SROC curve map were obtained to evaluate the diagnostic accuracy of the LAMP assay by applying Meta-Disc 1.4.0. The Stata 12.0 was used to draw Bivariate Boxplot, Deeks’ funnel plot, and Fagan Nomogram.

## Result

### Search results

The initial search retrieved 922 studies. After removing duplicates, 594 studies were retained, of which 526 studies were excluded because of the title and abstract. The remaining 68 studies were further read in full text, and finally, a total of 11 studies [3, 4, 6, 12–19] were included in our analysis (Additional file 1: Fig. S1).

### Characteristics of included study

We extracted a total of 19 sets of data from these 11 articles, which were summarized in Table 1.

**Table 1:** The detailed characteristics of the included studies

Author	Year	Study design	Country	Source of specimens	Gold standard	Type of LAMP	Bacterial species	TP	FP	FN	TN
Su	2014	Prospective	China	Clinical	Culture and latex agglutination^a^	orfX-LAMP	MRSA	557	0	9	101
Hanaki (a)	2011	Prospective	Japan	Clinical	PCR	LAMP	*S. aureus*	195	0	0	10
Hanaki (b)	2011	Prospective	Japan	Clinical	PCR	LAMP	MRSA	192	0	0	13
Metwally (a)	2014	Prospective	Egypt	Clinical	Standard procedures^b^	LAMP	*S. aureus*	36	0	0	24
Metwally (b)	2014	Prospective	Egypt	Clinical	Standard procedures^b^	LAMP	MRSA	19	0	0	41
Jiang (a)	2020	Prospective	China	Clinical	Culture^c^	m-LAMP-LFB	MRSA	8	0	0	88
Jiang (b)	2020	Prospective	China	Clinical	Culture^c^	m-LAMP-LFB	*S. aureus*	17	0	0	79
Kashani (a)	2020	Prospective	Iran	Clinical	Culture^d^	m-LAMP	*S. aureus*	49	0	0	4
Kashani (b)	2020	Prospective	Iran	Clinical	MIC and disk diffusion	m-LAMP	MRSA	37	4	0	12
Henares	2017	Prospective	Spain	Clinical	Culture^d^	eazyplex MRSA test system	*S. aureus*	5	1	1	44
Rödel (a)	2017	Prospective	Germany	Clinical	Routine species identification^d^	eazyplex® MRSA	*S. aureus*	31	2	0	106
Rödel (b)	2017	Prospective	Germany	Clinical	Routine species identification^d^	eazyplex® MRSA	MRSA	6	0	0	25
Nawattanapaiboon (a)^e^	2016	Prospective	Thailand	Clinical	Culture^f^	LAMP-LFD	MRSA	28	0	2	16
Nawattanapaiboon (b)	2016	Prospective	Thailand	Clinical	Culture^f^	LAMP-LFD	MRSA	52	0	0	20
Lim	2013	Prospective	Malaysia	Clinical	PCR	Loopamp DNA amplification kit	*S. aureus*	99	0	0	25
Leikeim (a)	2019	Retrospective	Germany	Clinical	Culture^g^	eazyplex® MRSA and eazyplex® MRSA plus test strips	*S. aureus*	230	5	1	561
Leikeim (b)	2019	Prospective	Germany	Clinical	Culture^g^	eazyplex® MRSA and eazyplex® MRSAplus	MRSA	32	2	0	763
Chen (a)	2020	Prospective	China	Clinical	Culture^h^	m-LAMP-LFB	*S. aureus*	28	0	0	35
Chen (b)	2020	Prospective	China	Clinical	Culture^h^	m-LAMP-LFB	MRSA	12	0	0	51

^a^Colony morphology, Gram staining, testing of catalase, hyaluronidase and coagulase, the Vitek 2 automated system and the API-Staph commercial kit

^b^Standard microbiological methods along with MecA PCR

^c^Culture, serum agglutination test and Gram stain

^d^The article is not described in detail

^e^It has two sets of data because they come from different samples

^f^Coagulase and antimicrobial susceptibility test

^g^Conventional blood culture diagnostics

^h^Blood culture, colony morphology, Gram staining, biochemical identification, and methicillin susceptibility testing

### Quality evaluation

**Fig. 1:**
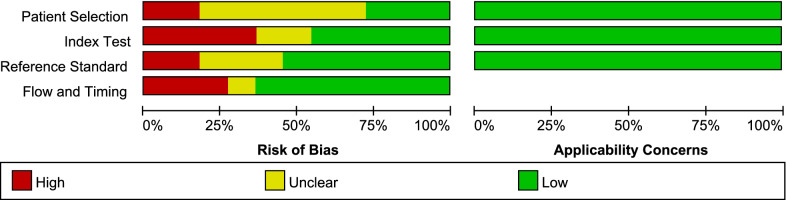
The summary of the risk of bias and applicability concerns of the included studies

**Fig. 2:**
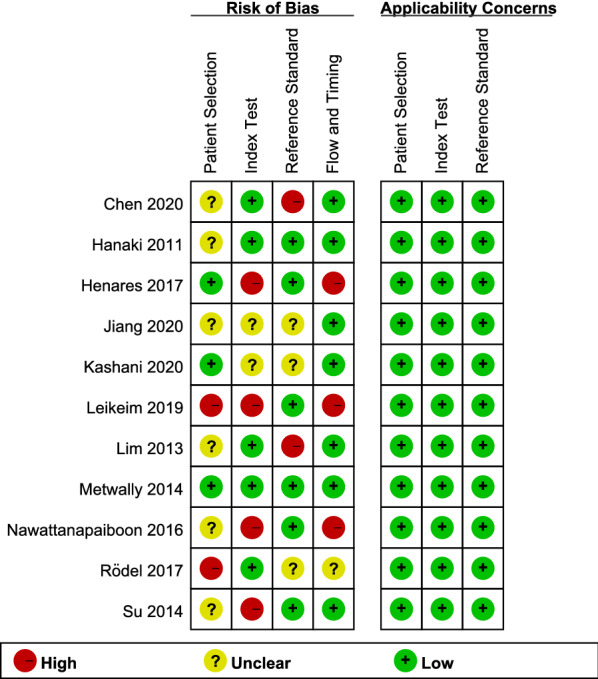
Quality evaluation of the individual studies

The quality plots were completed by using Review Manager 5.3.0 and were shown in Figs. 1 and 2. In the aspect of patient selection, 6 studies [3, 4, 6, 13, 15, 16] were considered as high risk of bias which was caused by didn’t avoid setting up a case–control trial. Furthermore, 3 studies [14, 17, 19] were evaluated as unclear risk of bias, because didn't mention whether improper exclusions had been avoided. In the aspect of the index test, 4 studies [4, 13, 16, 19] were considered as high risk of bias. Because the index test results were interpreted in a situation where the results of the gold standard were already known. In addition to this, the risk of bias was unclear for 2 studies [14, 18] because of the indistinct description. 2 studies [3, 6] which judged as having a high risk of bias in the domain of reference standard, known the results of LAMP assay when the index results were interpreted, while 3 studies [14, 15, 18] were unclear about it. Besides, 2 studies [3, 6] were considered as high risk of bias because didn’t have an appropriate interval of time, and only 1 study [15] was considered as unclear risk of bias because of the ambiguous description.

### Publication bias

**Fig. 3:**
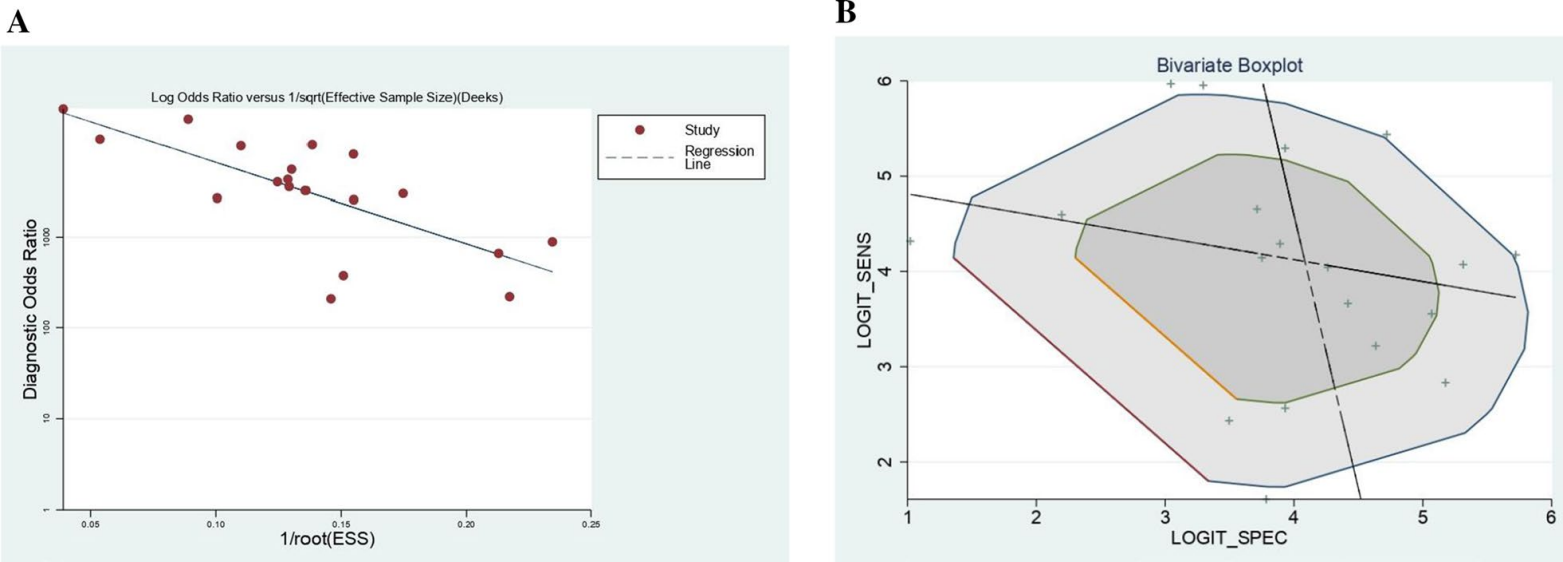
Deek’s funnel plot showing a significant publication bias (**A**), and Bivariate Boxplot indicating low heterogeneity (**B**)

According to the funnel plot (Fig. 3A), there was an existing significant publication bias in studies we included (P = 0.00).

Bivariate Boxplot showed that there was low heterogeneity in this systematic review (Fig. 3B).

### Diagnostic accuracy of the LAMP assay

**Fig. 4:**
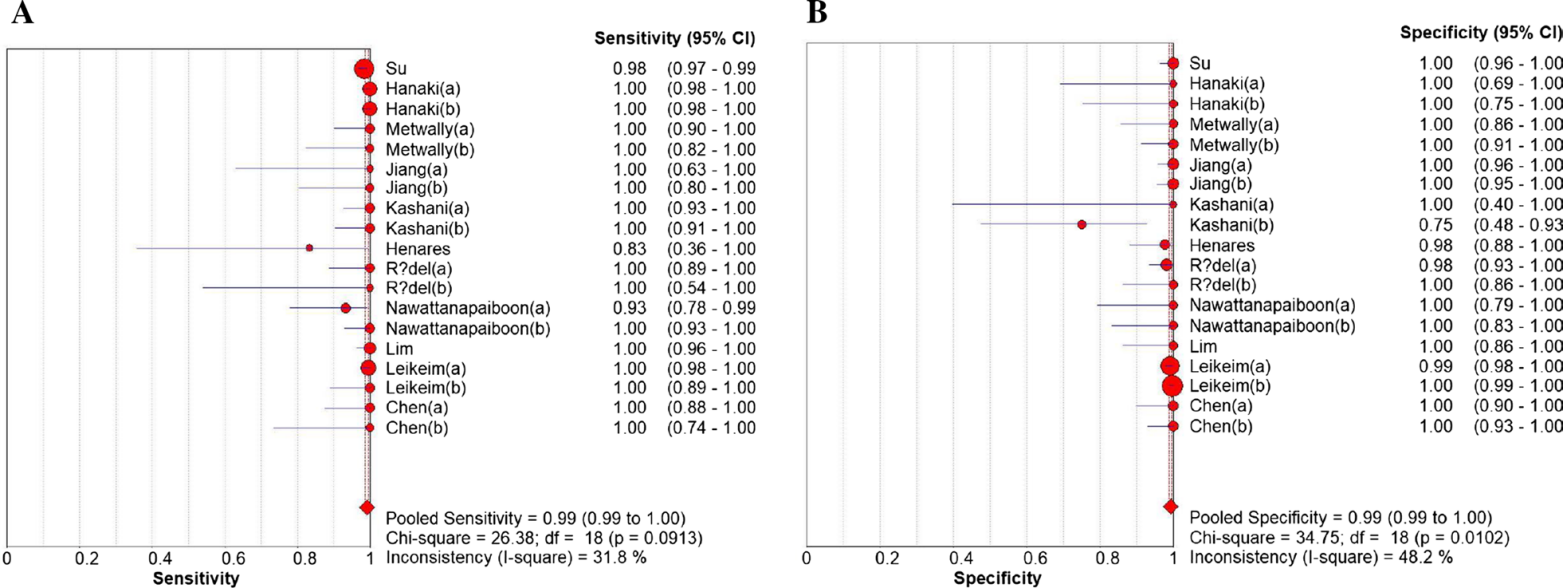
Forest plots for the pooled sensitivity (**A**) and specificity (**B**)

**Fig. 5:**
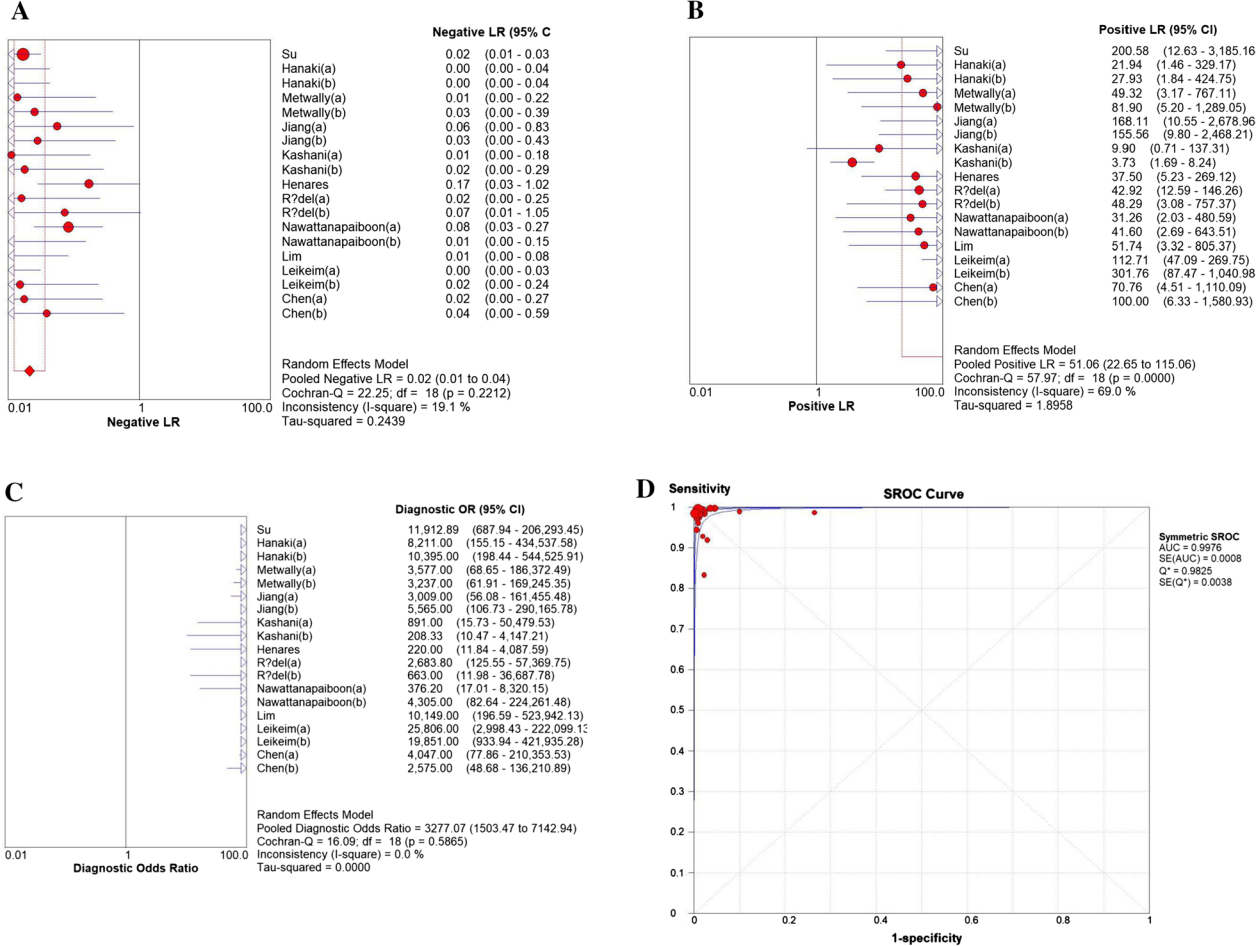
Negative LR (**A**), Positive LR (**B**), Diagnostic OR (**C**) and SROC curve (**D**) of the included studies

The sensitivity of LAMP ranged from 83 (95% CI 36–100) to 100%, whereas its pooled sensitivity in detecting *S. aureus* was 99% (95% CI 99–100), with the *I*-square value of 31.8% (Fig. 4A). Its specificity was ranged from 75 (95% CI 48–93) to 100%, whereas its pooled sensitivity was 99% (95% CI 99–100), with the *I*-square value of 48.2% (Fig. 4B). In addition to this, the NLR was 0.02 (95% CI 0.01 to 0.04, I^2^ = 19.1%; Fig. 5A), the PLR was 51.06 (95% CI 22.65 to 115.06, I^2^ = 69.0%; Fig. 5B), the DOR was 3277.07 (95% CI 1503.47 to 7142.94, I^2^ = 0.0%; Fig. 5C), and the AUC was 0.9976 (Fig. 5D).

**Fig. 6:**
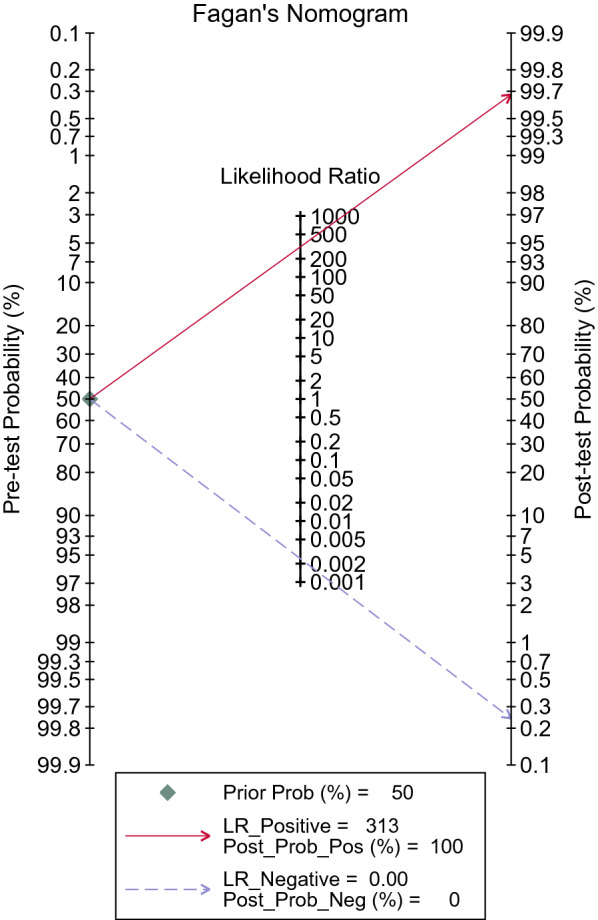
Fagan’s probability plot

The Fagan nomogram showed that with a prior probability of 50%, the post-test probability was 100% if the results were positive, and the post-test probability was 0% if the results were negative (Fig. 6).

### Diagnostic performance of subgroups

**Fig. 7:**
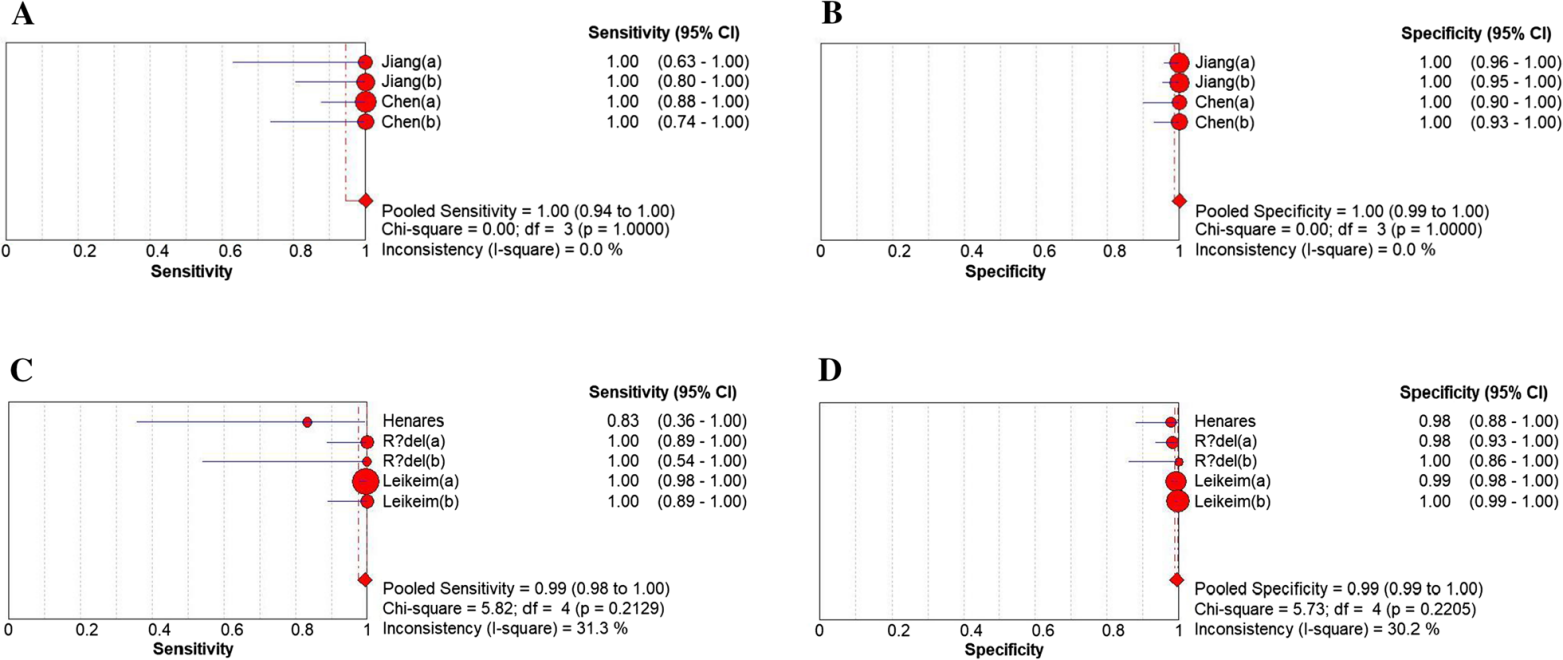
The results of subgroup analysis. **A**, **B** LAMP-LFB, **C**, **D** eazyplex^®^ MRSA

**Fig. 8:**
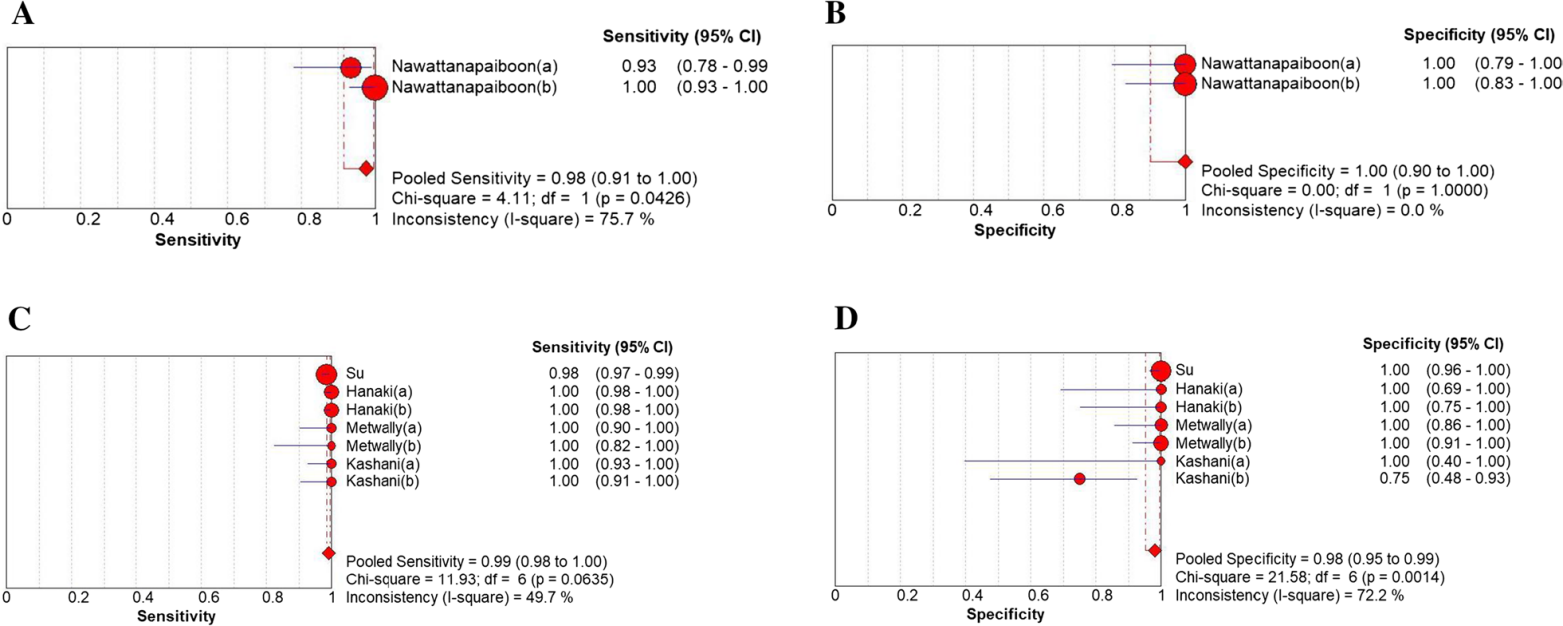
The results of subgroup analysis. **A**, **B** LAMP-LFD, (C, D) conventional LMAP

Two subgroups were generated in the study. The first subgroup analysis was performed to better distinguish the diagnostic effects of different LAMP types (Figs. 7, 8). In the subgroup, the lamp-FLB and Loopamp DNA Amplification kit have the highest diagnostic accuracy for positive samples (100%, but the latter study only has a four-lattice table). This is followed by eazyplex^®^ MRSA and LAMP (studies that did not indicate LAMP classification were summarized as LAMP groups) (99%) and LAMP-FLD (98%). In terms of diagnostic accuracy for negative samples, from high to low are lamp-FLB, lamp-FLD, and Loopamp DNA Amplification kit (100%), eazyplex^®^ MRSA (99%), and LAMP (98%), respectively.

Another subgroup analysis was performed according to the type of gold standards. In the result, compared with PCR, the sensitivity and specificity were both 100%. The sensitivity and specificity were both 99%, when compared with traditional identification methods.

## Discussion

In the study, we explored the diagnostic efficacy of LAMP for *S. aureus* by systematic review and meta-analysis. High diagnostic performance of LAMP in the detection of *S. aureus* was revealed in our study.

In both positive and negative samples, high sensitivity and specificity were evaluated in the study. Among them, there were only 2 studies (4 data in) with low sensitivity and specificity (both < 60% CI). Henares’ study [16] had a minimum sensitivity of 0.36, and Rödel’s study [15] had a minimum sensitivity of 0.54, respectively. In specificity, in Kashani’s study [14] low diagnostic performance was shown, with 0.40 and 0.48 when low. For these 4 data sets, low levels may be owing to two reasons: On the one hand, the small sample size was used in Henares’ study [16]. On the other hand, it should be noticed that MIC and Disk Diffusion were used as the gold standards in Kashani’s study [14], but there is little literature on this diagnostic method as the gold standard in.

In the first subgroup analysis, the best diagnostic performance was found when used m-LAMP-LFB (both sensitivity and specificity reached 100%, short for multiplex loop-mediated isothermal amplification linked to a nanoparticle-based lateral flow biosensor), which uses FLB to interpret LAMP diagnosis results. Compared with other monitoring techniques such as gel electrophoresis and real-time turbidity, LFB is specific for target genes and less likely to result in false–positive results. Besides, it’s effective and quick to be operated [6, 18]. Another subgroup analysis reveals that high diagnostic performance exists whatever the type of goal standards. Although both sensitivity and specificity were higher in LAMP when compared with PCR assay (100%), the difference between PCR and other assays (99%) was mostly negligible.

With a PLR of 51.06 and an NLR of 0.02 in summary, which was classified that LAMP can guarantee higher TP and TN while avoiding FP and FN. In addition, when the prior probability of 50%, LAMP could diagnose with a posterior sensitivity of 100%, and a posterior specificity of 0% in Fagan’s plot.

In general, good diagnostic performance was shown in LAMP in the detection *S. aureus*. Among them, LAMP-FLD has the best overall diagnostic performance of both positive and negative samples according to comprehensive judgment.

In the part of publication bias, there are many points asymmetrically distributed in Deek’s funnel plot, the result in it is a collection with Publication bias score from Cochrane. Some reasons may lead to this result: (1) Many discontinuous or nonrandomized cases were included in the study; (2) A case–control study design was used; (3) In terms of the application of the blinding method, LAMP results were interpreted under the known gold standard results in many studies; (4) The time interval of LAMP and Gold standard interpretation is ambiguous. These factors may have resulted in a "beautiful" experimental result, but they also led to a significant publication bias in our study [20]. Therefore, although we believe that LAMP has good diagnostic performance, the significant publication bias in the literature included in this systematic review leads us to remain cautious about the results.

In terms of heterogeneity, the results of I-square in forest plots of sensitivity, specificity, and diagnosability show no heterogeneity in our study. Moreover, the Bivariate Boxplot also confirms the results of these I-square from 3 forest plots. Thus, we assess there is no heterogeneity in our study.

Overall, our study demonstrates the efficacy of LAMP in the diagnosis of *S. aureus* by combining previously published studies. In addition, since there is no systematic review of this area, our study certainly fills the gap.

There are also some shortcomings in this study. On the one hand, only English studies were included in the study. On the other hand, in the subgroup analysis, although we found that all kinds of LAMP had good diagnostic performance, the number of samples in the subgroup was not enough, and there were only a few pieces of data in each subgroup. If confirmed by more clinical studies, the results would be more reliable.

## Conclusion

In summary, our study shows that the LAMP assay has high diagnostic accuracy in the diagnosis of *S. aureus*. Nevertheless, more research is necessary to determine the diagnostic accuracy of LAMP assay.

## Supplementary Information

**Additional file1: Figure S1.** Flow chart for the selection of studies.

## Data Availability

All data analyzed in this study are included in the article.
