# Supplementary material for: Evaluation of the loop-mediated isothermal amplification assay for Staphylococcus aureus detection: a systematic review and meta-analysis
Source: Ann Clin Microbiol Antimicrob. 2022 Jun 24;21:27. doi: 10.1186/s12941-022-00522-6 (PMC9233341; doi:10.1186/s12941-022-00522-6)
Supplement: Supplementary file 1 — Additional file1: Figure S1. Flow chart for the selection of studies. [file 12941_2022_522_MOESM1_ESM.docx]

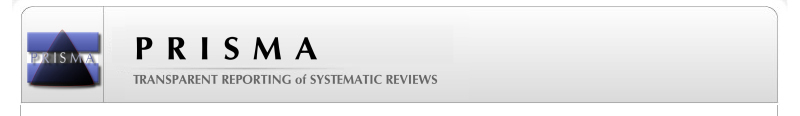
PRISMA 2009 Flow Diagram

Studies included in quantitative synthesis (meta-analysis)
(n =11)

Studies included in qualitative synthesis
(n = 31)

Records screened
(n =594)

Records after duplicates removed
(n = 594)

## Identification

## Eligibility

## Included

## Screening

Records excluded
(n = 526)

Full-text articles excluded, with reasons
(n =20)

19 studies' data could not form into a 2*2 table

1 inappropriate reference standard

Full-text articles excluded, with reasons
(n =37)

4 excluded by no full text

32 not samples from human

1 inappropriate reference standard

Additional records identified through other sources
(n =0 )

Pubmed (252)

Embase (169)

Cochrane Library (8)

Web of science (493)
(n =914 )

Full-text articles assessed for eligibility
(n =68)
